# Chronic Granulomatous Fungal Rhinosinusitis Mimicking a Giant Cell Tumor of the Maxilla: A Diagnostic Pitfall

**DOI:** 10.7759/cureus.107044

**Published:** 2026-04-14

**Authors:** Saikat Mitra, Neelesh Shrivastava

**Affiliations:** 1 Pathology, All India Institute of Medical Sciences Nagpur, Nagpur, IND; 2 Surgical Oncology, All India Institute of Medical Sciences Nagpur, Nagpur, IND

**Keywords:** aspergillus, central giant cell lesions, chronic granulomatous fungal rhinosinusitis, giant-cell tumor of bone, invasive fungal sinusitis, peripheral giant cell granuloma, suppurative granuloma, total maxillectomy

## Abstract

Chronic granulomatous fungal rhinosinusitis is an uncommon form of invasive fungal sinusitis that typically affects immunocompetent individuals and often presents as a slowly progressive mass lesion, closely mimicking malignancy clinically and radiologically. We report the case of a 56-year-old woman who presented with a painless swelling over the left maxillary region for four months. She had a history of a similar lesion at the same site nine years earlier, which had been surgically excised elsewhere and diagnosed as a giant cell tumor of the maxilla. Magnetic resonance imaging revealed an expansile lesion involving the left maxillary sinus with extension into adjacent facial soft tissues and orbital contents, raising suspicion for a neoplastic process. Fine-needle aspiration cytology demonstrated numerous multinucleated giant cells, suggesting a giant cell tumor. The patient underwent total maxillectomy with orbital exenteration. Histopathological examination revealed well-formed non-caseating granulomas with central neutrophilic microabscesses forming suppurative granulomas. Slender septate fungal hyphae with acute-angle branching were identified on special stains, consistent with *Aspergillus* species. A final diagnosis of chronic granulomatous fungal rhinosinusitis was established. The patient was treated with intravenous voriconazole postoperatively and remains clinically stable on follow-up. This case highlights the importance of considering invasive fungal sinusitis in the differential diagnosis of destructive maxillofacial masses and underscores the critical role of histopathological evaluation in preventing misdiagnosis and unnecessary radical surgery.

## Introduction

Fungal rhinosinusitis encompasses a heterogeneous group of disorders ranging from non-invasive colonization to aggressive, life-threatening invasive infections [1]. Based on clinical course, host immune status, and histopathological features, fungal rhinosinusitis is broadly classified into non-invasive and invasive forms. Invasive fungal rhinosinusitis is further subdivided into acute invasive, chronic invasive, and granulomatous invasive variants [2]. Although relatively uncommon overall, the granulomatous subtype shows a higher prevalence in certain geographic regions, particularly South Asia, the Middle East, and parts of Africa, suggesting an environmental and climatic influence on disease distribution [1].

Chronic granulomatous fungal rhinosinusitis (CGFRS) represents a distinct and relatively uncommon subtype of invasive fungal disease that predominantly affects immunocompetent individuals [3]. It is characterized histologically by well-formed granulomas composed of epithelioid histiocytes and multinucleated giant cells, associated fibrosis, and typically sparse fungal hyphae, most commonly due to *Aspergillus* species. Unlike acute invasive fungal sinusitis, which progresses rapidly and is frequently angio-invasive, the granulomatous form follows an indolent but locally destructive course.

Clinically and radiologically, CGFRS often presents as a slowly enlarging mass with bone remodeling or erosion and extension into adjacent soft tissues. These features can closely simulate benign or malignant neoplasms of the maxillofacial region, posing significant diagnostic challenges in routine clinical practice - particularly in small biopsies or cytology specimens where fungal elements may be sparse or easily overlooked. Such misinterpretation can lead to inappropriate clinical decision-making and, in some cases, unnecessarily radical surgical interventions with significant morbidity.

Recognition of its characteristic histopathological pattern, particularly suppurative granulomas with sparse fungal elements, is therefore essential for accurate diagnosis and appropriate management.

We report a case of recurrent chronic granulomatous fungal rhinosinusitis presenting as a tumor-like maxillary mass with orbital involvement, initially misdiagnosed as a giant cell tumor, highlighting an important diagnostic pitfall.

## Case presentation

A 56-year-old woman with no known comorbidities presented with a painless swelling over the left side of the face for four months. The swelling was gradually progressive, resulting in facial asymmetry. There was no history of fever, nasal discharge, epistaxis, visual disturbance, or weight loss. There was no significant occupational exposure to organic dust, farming, or construction work, and no history of prolonged environmental exposure to decaying vegetation or soil. The patient also denied any prior history of chronic sinusitis, nasal allergies, or immunosuppressive conditions.

The patient reported a similar swelling at the same site nine years earlier, for which she had undergone surgery at an outside center. At that time, the lesion was diagnosed as a giant cell tumor of the maxilla. However, detailed records of prior radiological findings or operative procedures were unavailable to us. The histology slides and blocks could not be retrieved from the outside center for review.

On local examination, the swelling over the left midface measured approximately 6×5 cm, was firm in consistency, non-tender, and had ill-defined margins. The overlying skin appeared stretched but intact, with no ulceration or discoloration. The swelling was relatively fixed to underlying structures with minimal mobility. Intraoral examination revealed fullness of the left buccal sulcus without mucosal breach. There was no active nasal discharge or visible intranasal mass on anterior rhinoscopy. Ocular examination revealed mild proptosis with inferior displacement of the globe; however, visual acuity and extraocular movements were preserved. No cervical lymphadenopathy was identified. Routine laboratory investigations, including complete blood count, renal and liver function tests, and blood glucose levels, were within normal limits. There was no evidence of immunosuppression. Inflammatory markers were not significantly elevated. Serological tests for fungal infection were not performed preoperatively.

**Figure 1:**
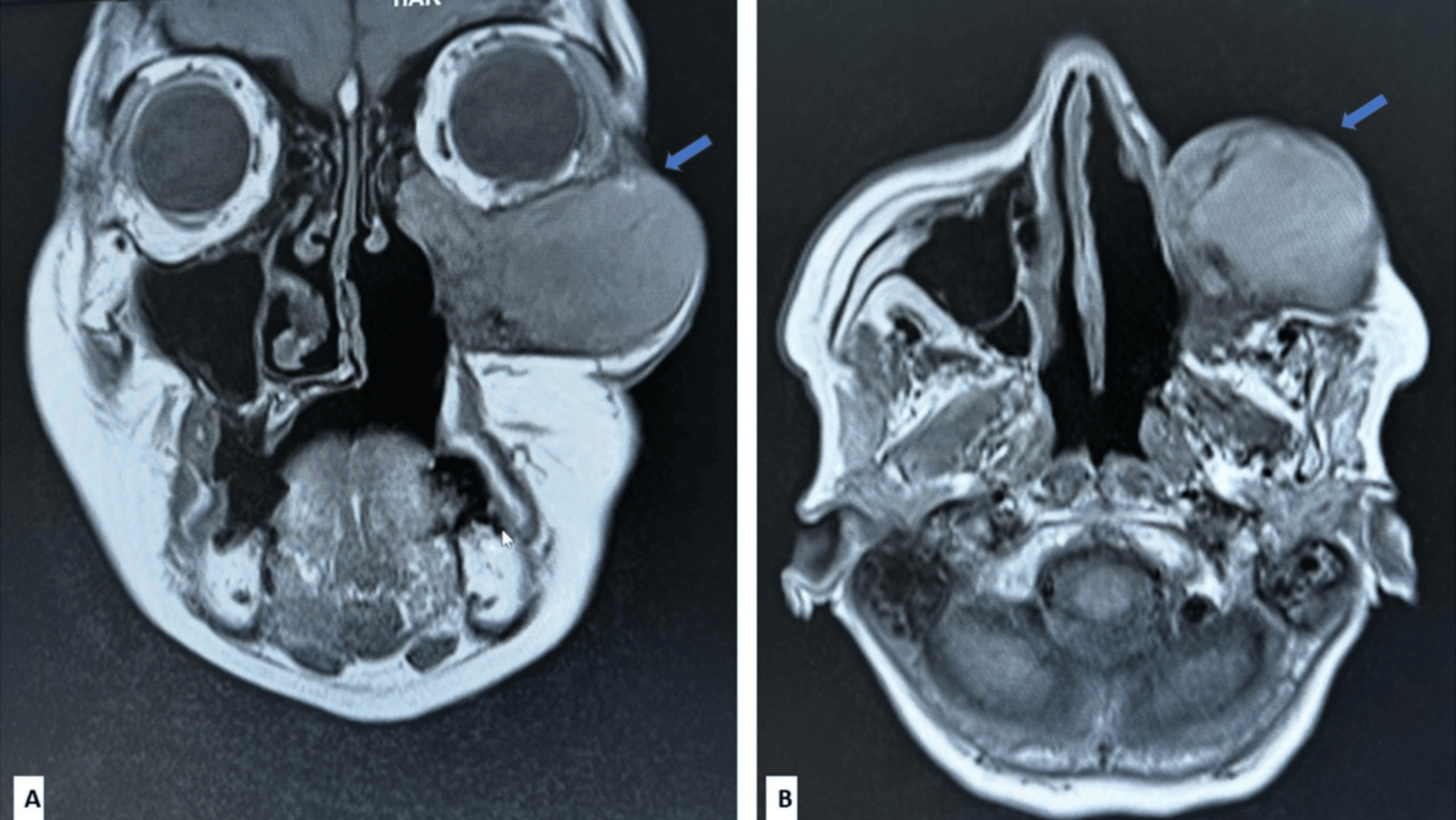
Magnetic resonance imaging (MRI) of the paranasal sinuses and orbit Magnetic resonance imaging (MRI) of the paranasal sinuses and orbit demonstrating a mass-forming lesion. A. Coronal section shows a heterogeneously intense soft-tissue mass occupying the maxillary sinus with extension into the adjacent nasal cavity and infraorbital region, causing facial asymmetry (blue arrow). B. Axial section reveals the lesion abutting the orbital floor with mass effect on the orbit, mimicking a neoplastic process (blue arrow).

Magnetic resonance imaging (MRI) of the paranasal sinuses demonstrated a well-defined expansile lesion centered in the left maxillary sinus. The mass appeared markedly hyperintense on T2-weighted images and showed remodeling and thinning of the sinus walls. Extension into adjacent facial soft tissues and the orbital cavity was noted, raising suspicion for a neoplastic process. The MRI findings are highlighted in Figure 1. 

Fine-needle aspiration cytology from the facial swelling revealed numerous multinucleated giant cells in a pauci-inflammatory background, leading to a provisional diagnosis of a giant cell tumor. Based on the clinical and radiological findings, a provisional diagnosis of a neoplastic process was considered. The differential diagnoses included giant cell tumor/central giant cell granuloma, and other expansile lesions of the maxilla. In view of the radiological appearance and cytological findings showing numerous multinucleated giant cells, a giant cell tumor was favored preoperatively.

The patient underwent wide local excision in the form of total maxillectomy with orbital exenteration. Intraoperatively, a well-defined but locally infiltrative mass was identified involving the left maxillary sinus, with extension into adjacent facial soft tissues and the orbital cavity. The lesion was firm to hard in consistency and showed areas of adherence to surrounding structures, necessitating en-bloc resection. The orbital contents were sacrificed due to close proximity and involvement of the orbital floor, with concern for incomplete clearance if preserved. Adequate margins were attempted intraoperatively, and hemostasis was achieved. The resected specimen was sent for histopathological evaluation.

**Figure 2:**
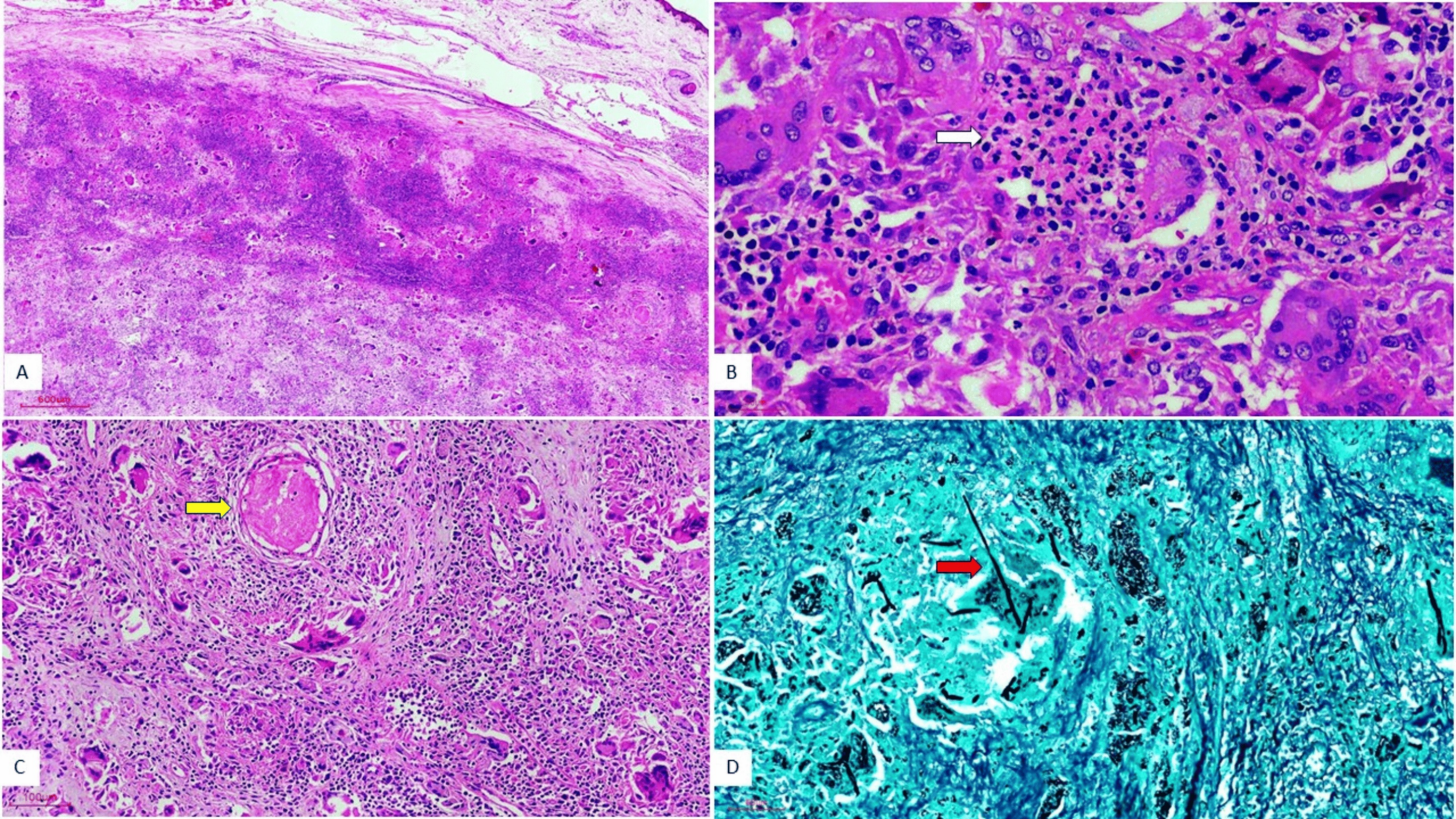
Histopathology findings from the excised mass (A) (Hematoxylin and eosin (H&E), 200x): Section shows well-circumscribed granulomatous inflammation admixed with giant cells and fibrosis. (B) (H&E, 400x): Section demonstrating suppurative granuloma characterized by central neutrophilic microabscess formation (white arrow) surrounded by epithelioid histiocytes and inflammatory cells. (C) (H&E, 200x): Section showing prominent perineural granulomatous inflammation (yellow arrow) with surrounding fibrosis, indicative of invasive disease. (D) (Gomori methenamine silver (GMS), 200x): Section highlighting slender, septate fungal hyphae with acute-angle branching (red arrow), consistent with *Aspergillus* species. GMS stain highlights fungal cell walls in black.

Histopathological examination revealed multiple well-formed non-caseating granulomas composed of epithelioid histiocytes and numerous multinucleated giant cells within a fibrotic stroma. Several granulomas demonstrated central aggregates of neutrophils forming suppurative granulomas (Figure 2A, 2B). Prominent perineural granulomatous inflammation with associated fibrosis was also identified (Figure 2C). On careful examination, slender septate fungal hyphae with acute-angle branching were identified within the granulomas and surrounding tissue. Special stains, including Periodic acid-Schiff (PAS) and Grocott methenamine silver (GMS), were performed to enhance visualization of fungal elements, particularly in areas with dense granulomatous inflammation Figure 2D), consistent with *Aspergillus* species. The granulomatous inflammation infiltrated adjacent facial bones and ocular muscles. The histopathology findings are highlighted in Figure 2.

**Figure 3:**
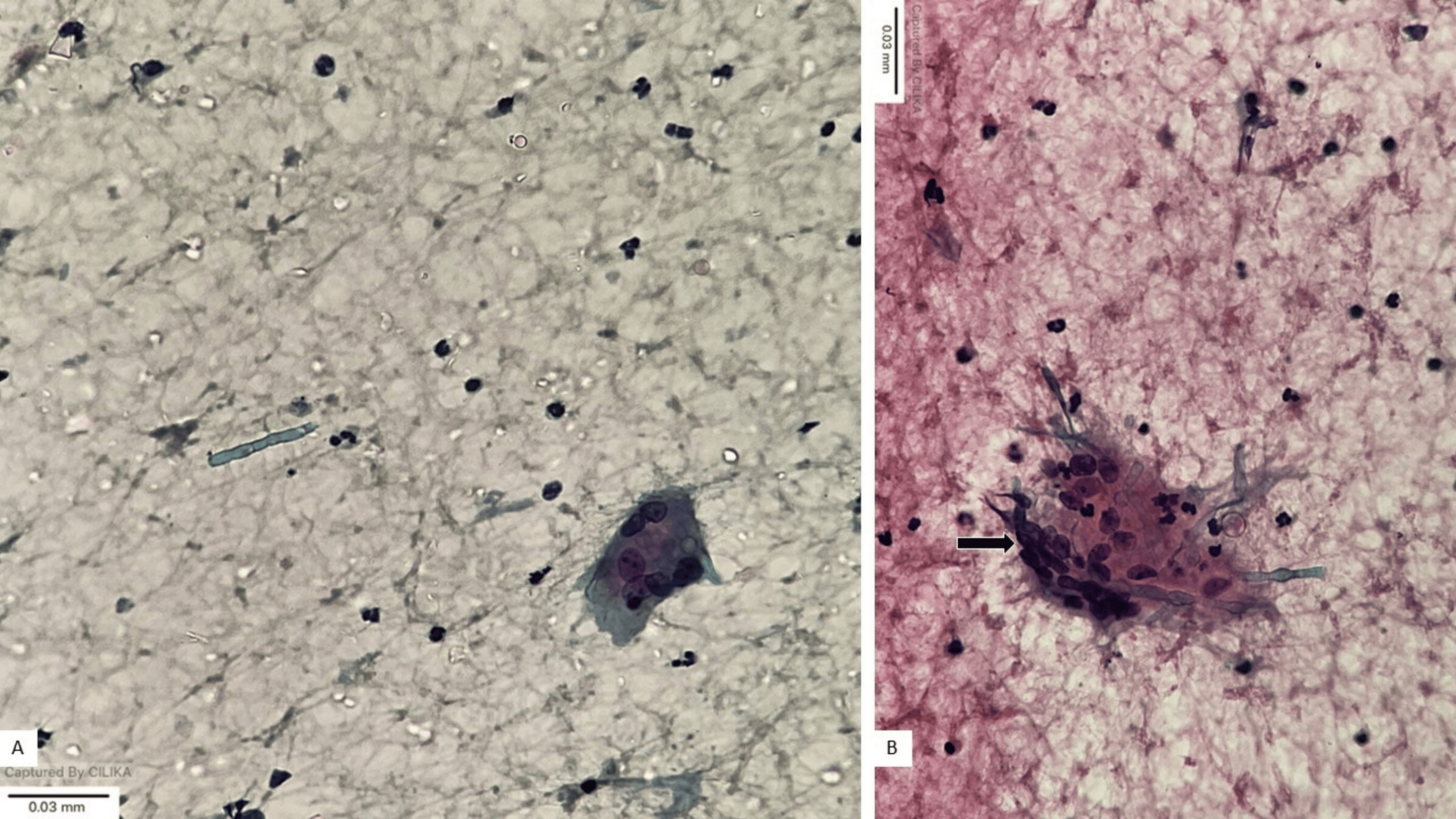
Fine-needle aspiration cytology (FNAC) smear (A) (Papanicolaou (PAP), 100x): Low-power view showing a cellular smear with scattered inflammatory cells along with occasional multinucleated giant cells. (B) (PAP, 400x)- Higher magnification demonstrating a multinucleated giant cell (arrow) with engulfed slender, septate fungal hyphae exhibiting acute-angle branching. The surrounding background shows inflammatory cells and debris.

A review of the cytology slides revealed numerous multinucleated giant cells, some containing engulfed fungal profiles that had not been recognized at the time of initial diagnosis (Figure 3).

Based on the histopathological findings, a diagnosis of chronic granulomatous fungal rhinosinusitis due to *Aspergillus* species was established. Postoperatively, the patient was treated with intravenous voriconazole (6 mg/kg once every 12 hours on day 1, then 4 mg/kg once every 12 hours for 7 days). Later, she was switched to oral voriconazole 200 mg once every 12 hours for 14 days. She was discharged after 15 days of surgery in a clinically stable condition. She remained asymptomatic after six months of follow-up without any clinical or imaging evidence of recurrence.

## Discussion

Chronic granulomatous fungal rhinosinusitis represents an uncommon but distinctive form of invasive fungal sinusitis that predominantly affects immunocompetent individuals and follows an indolent yet locally destructive course [2,4]. Because of its slow progression and mass-forming behavior, it frequently mimics benign or malignant neoplasms of the maxillofacial region, resulting in diagnostic delays and, at times, unnecessarily aggressive surgical management.

The classification of fungal rhinosinusitis remains an area of ongoing discussion. According to the widely accepted schema proposed by Chakrabarti et al., invasive fungal rhinosinusitis is categorized into acute invasive, chronic invasive, and granulomatous invasive forms based on clinical duration, host immune status, and histomorphology [2]. Chronic granulomatous fungal rhinosinusitis is characterized by well-formed granulomas, multinucleated giant cells, fibrosis, and sparse fungal hyphae, most commonly due to *Aspergillus* species. However, overlap between chronic invasive and granulomatous invasive forms has been reported, with granulomatous inflammation occasionally observed in cases classified as chronic invasive fungal rhinosinusitis [5,6]. This overlap underscores the importance of clinicopathological correlation rather than rigid reliance on histological subtyping alone.

Among the histopathological features, the presence of suppurative granuloma, that is, well-formed granulomas with central neutrophilic microabscess, has been described as highly suggestive and, in some reports, pathognomonic of the granulomatous form [5-7]. In the present case, identification of suppurative granulomas prompted a meticulous search for fungal elements, which were otherwise sparse and could easily have been overlooked on routine hematoxylin and eosin sections. This emphasizes the practical importance of recognizing characteristic inflammatory patterns, particularly when fungal organisms are scant.

Perineural granulomatous inflammation, as observed in our patient, further supports an invasive fungal etiology. Although vascular invasion is more commonly associated with acute invasive disease, chronic forms may demonstrate perineural spread and adjacent soft-tissue infiltration, reflecting their locally aggressive behavior [4-6].

Clinically and radiologically, chronic granulomatous fungal rhinosinusitis may present as a destructive sinonasal mass with bone erosion and orbital or intracranial extension, closely simulating malignancy. Several case reports have documented similar presentations mimicking sinonasal carcinoma, sarcoma, or sellar tumors [7-9]. Central giant cell granuloma, a benign, nonodontogenic lesion of the jaw of osteoclastic origin, is commonly identified in the mandible as well as maxilla and can form lobulated sclerotic to osteolytic mass and show local destruction and clinically aggressive behavior [10]. In our case, both imaging and fine-needle aspiration cytology favored a giant cell tumor, and the previous history of a similarly diagnosed lesion further reinforced the neoplastic impression. Only detailed histopathological examination with special stains established the correct diagnosis.

Another important consideration is the spectrum of causative organisms. While *Aspergillus* species remain the most frequently implicated pathogens in chronic granulomatous fungal rhinosinusitis, regional variations and occasional involvement of other fungi have been reported [4,7]. The relative paucity of fungal elements in tissue sections may contribute to under-recognition, particularly in small biopsies or cytology specimens. In addition to conventional histopathological examination and special stains, newer diagnostic modalities such as polymerase chain reaction (PCR)-based fungal detection and sequencing techniques have shown promise in improving diagnostic sensitivity, particularly in cases with scant fungal elements. These molecular methods can facilitate rapid and species-specific identification, even in formalin-fixed paraffin-embedded tissue. However, their availability remains limited in routine practice, and they should be considered complementary rather than a replacement for careful morphological assessment.

The choice of antifungal therapy in chronic granulomatous fungal rhinosinusitis is guided by the most commonly implicated organisms. Voriconazole is considered the first-line agent due to its excellent activity against *Aspergillus*, favorable tissue penetration, and established efficacy in invasive fungal infections. The duration of therapy, although not standardized, is typically individualized based on disease extent, completeness of surgical excision, and clinical response. In the present case, a short course of intravenous therapy followed by oral voriconazole was administered, given the absence of systemic dissemination and satisfactory surgical clearance.

This case highlights several diagnostic pitfalls. Abundant multinucleated giant cells on cytology and prior biopsy led to an initial diagnosis of a giant cell tumor, illustrating how inflammatory granulomatous processes may mimic primary bone or soft tissue neoplasms. Careful evaluation for suppurative granulomas and judicious use of special stains are therefore essential when assessing destructive maxillofacial masses, even in immunocompetent patients.

## Conclusions

Chronic granulomatous fungal rhinosinusitis should be considered in the differential diagnosis of slowly progressive, destructive maxillofacial masses, even in immunocompetent individuals. Its indolent course, mass-forming presentation, and radiological resemblance to neoplastic lesions can lead to misdiagnosis and, as illustrated in the present case, potentially result in unnecessarily radical surgical interventions associated with significant functional and cosmetic morbidity.

Recognition of characteristic histopathological features, particularly well-formed granulomas with central neutrophilic microabscesses forming suppurative granulomas, is essential for raising suspicion of an underlying fungal etiology. Careful evaluation for sparse fungal elements using appropriate special stains plays a critical role in establishing the correct diagnosis and guiding appropriate therapy.

Greater awareness of this entity, along with a high index of suspicion and close clinicopathological correlation, is essential to avoid diagnostic pitfalls. A multidisciplinary approach involving clinicians, radiologists, and pathologists can further enhance diagnostic accuracy, facilitate timely initiation of antifungal therapy, and ultimately improve patient outcomes while preventing avoidable aggressive surgical management.
